# Introducing the Outbreak Threshold in Epidemiology

**DOI:** 10.1371/journal.ppat.1003277

**Published:** 2013-06-06

**Authors:** Matthew Hartfield, Samuel Alizon

**Affiliations:** Laboratoire MIVEGEC (UMR CNRS 5290, IRD 224, UM1, UM2), Montpellier, France; (The Fox Chase Cancer Center, United States of America)

## Abstract

When a pathogen is rare in a host population, there is a chance that it will die out because of stochastic effects instead of causing a major epidemic. Yet no criteria exist to determine when the pathogen increases to a risky level, from which it has a large chance of dying out, to when a major outbreak is almost certain. We introduce such an outbreak threshold (*T_0_*), and find that for large and homogeneous host populations, in which the pathogen has a reproductive ratio *R_0_*, on the order of 1/Log(*R_0_*) infected individuals are needed to prevent stochastic fade-out during the early stages of an epidemic. We also show how this threshold scales with higher heterogeneity and *R_0_* in the host population. These results have implications for controlling emerging and re-emerging pathogens.

With the constant risk of pathogens emerging [1], such as Severe Acute Respiratory Syndrome (SARS) or avian influenza virus in humans, foot-and-mouth disease virus in cattle in the United Kingdom [2], or various plant pathogens [3], it is imperative to understand how novel strains gain their initial foothold at the onset of an epidemic. Despite this importance, it has seldom been addressed how many infected individuals are needed to declare that an outbreak is occurring: that is, when the pathogen can go extinct due to stochastic effects, to when it infects a high enough number of hosts such that the outbreak size increases in a deterministic manner (Figure 1A). Generally, the presence of a single infected individual is not sufficient to be classified as an outbreak, so how many infected individuals need to be present to cause this deterministic increase? Understanding at what point this change arises is key in preventing and controlling nascent outbreaks as they are detected, as well as determining the best course of action for prevention or treatment.

**Figure 1:**
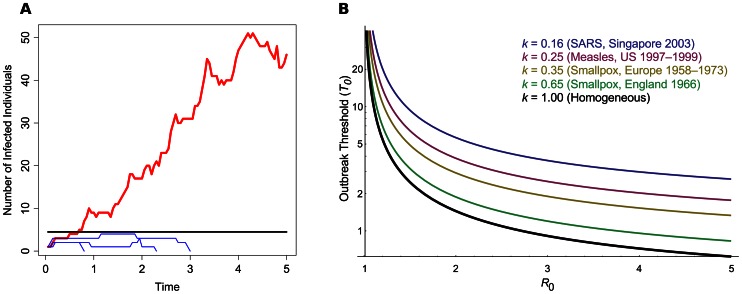
The outbreak threshold in homogeneous and heterogeneous populations. (**A**) A schematic of pathogen emergence. This graph shows the early stages of several strains of an epidemic, where *R_0_* = 1.25. The black line denotes the outbreak threshold (*T*_0_ = 1/Log(*R_0_*) = 4.48). Blue thin lines show cases in which the pathogen goes extinct and does not exceed the threshold; the red thick line shows an epidemic that exceeds the threshold and persists for a long period of time. Simulations were based on the Gillespie algorithm [22]. (**B**) Outbreak threshold in a homogeneous (black thick line) or in a heterogeneous population, for increasing *R_0_*. The threshold was calculated following the method described by Lloyd-Smith et al. [11] and is shown for different values of *k*, the dispersion parameter of the offspring distribution, as obtained from data on previous epidemics [11]. If the threshold lies below one, this means that around only one infected individual is needed to give a high outbreak probability.

The classic prediction for pathogen outbreak is that the pathogen's reproductive ratio (*R_0_*), the number of secondary infections caused by an infected host in a susceptible population, has to exceed one [4], [5]. This criterion only strictly holds in deterministic (infinite population) models; in finite populations, there is still a chance that the infection will go extinct by chance rather than sustain itself [4]–[6]. Existing studies usually consider random drift affecting outbreaks in the context of estimating how large a host population needs to be to sustain an epidemic (the “Critical Community Size” [4], [7], [8]), calculating the outbreak probability in general [9]–[12], or ascertaining whether a sustained increase in cases over an area has occurred [13]. Here we discuss the fundamental question of how many infected individuals are needed to almost guarantee that a pathogen will cause an outbreak, as opposed to the population size needed to maintain an epidemic once it has appeared (Critical Community Size; see also Box 1). We find that only a small number of infected individuals are often needed to ensure that an epidemic will spread.

**Box 1. Glossary of Key Terms**

- The **Basic Reproductive Ratio** (*R_0_*) is the number of secondary infections caused by a single infected individual, in a susceptible population. It is classically used to measure the rate of pathogen spread. In infinite-population models, a pathogen can emerge if *R_0_*>1. In a finite population, the pathogen can emerge from a single infection with probability 1-1/*R_0_* if *R_0_*>1, otherwise extinction is certain.
- The **Critical Community Size** (CCS) is defined as the total population size (of susceptible and infected individuals, or others) needed to sustain an outbreak once it has appeared. This idea was classically applied to determining what towns were most likely to maintain measles epidemics [7], so that there would always be some infected individuals present, unless intervention measures were taken.
- The **Outbreak Threshold** (*T_0_*) has a similar definition to the CSS, but is instead for use at the onset of an outbreak, rather than once it has appeared. It measures how many infected individuals (not the total population size) are needed to ensure that an outbreak is very unlikely to go extinct by drift. Note that the outbreak can still go extinct in the long term, even if *T_0_* is exceeded, if there are not enough susceptible individuals present to carry the infection afterwards.

We introduce the concept of the outbreak threshold (denoted *T_0_*), which we define as the number of infected individuals needed for the disease to spread in an approximately deterministic manner. *T_0_* can be given by simple equations. Indeed, if the host population is homogeneous (that is, where there is no individual variability in reproductive rates) and large enough so that depletion of the pool of susceptible hosts is negligible, then the probability of pathogen extinction if *I* infected hosts are present is (1/*R_0_*)*^I^* ([6], details in Material S.1 in Text S1). By solving this equation in the limit of extinction probability going to zero, we find that on the order of 1/Log(*R_0_*) infected hosts are needed for an outbreak to be likely (black thick curve in Figure 1B), a result that reflects similar theory from population genetics [14]–[16]. Note that this result only holds in a finite population, as an outbreak in a fully susceptible infinite population is certain if *R_0_*>1 ([4], see also Material S.1 in Text S1).

This basic result can be modified to consider more realistic or precise cases, and *T_0_* can be scaled up if an exact outbreak risk is desired. For example, for the pathogen extinction probability to be less than 1%, there needs to be at least 5/Log(*R_0_*) infected individuals. More generally, the pathogen extinction probability is lower than a given threshold *c* if there are at least −Log(*c*)/Log(*R_0_*) infected individuals. Furthermore, if only a proportion *p*<1 of all infected individuals are detected, then the outbreak threshold order is *p*/Log(*R_0_*). Also, if there exists a time-lag *τ* between an infection occurring and its report, then the order of *T_0_* is *e^−τ(β-δ)^*/Log(*R_0_*), where *β* is the infection transmission rate and 1/*δ* the mean duration of the infectious period (Material S.1 in Text S1). Finally, we can estimate how long it would take, on average, for the threshold to be reached and find that, if the depletion in susceptible hosts is negligible, this duration is on the order of 1/(*β-δ)* (Material S.1 in Text S1).

So far we have only considered homogenous outbreaks, where on average each individual has the same pathogen transmission rate. In reality, there will be a large variance among individual transmission rates, especially if “super-spreaders” are present [17]. This population heterogeneity can either be deterministic, due to differences in immune history among hosts or differences in host behavior, or stochastic, due to sudden environmental or social changes. Spatial structure can also act as a form of heterogeneity, if each region or infected individual is subject to different transmission rates, or degree of contact with other individuals [18]. In such heterogeneous host populations, the number of secondary cases an infected individual engenders is jointly captured by *R_0_* and a dispersion parameter *k* (see Box 2). This dispersion parameter controls the degree of variation in individual transmission rates, while fixing the average *R_0_*. The consequence of this model is that the majority of infected hosts tend to cause few secondary infections, while the minority behave as super-spreaders, causing many secondary infections. Host population heterogeneity (obtained with lower values of *k*) increases the probability that an outbreak will go extinct, as the pathogen can only really spread via one of the dwindling super-spreading individuals. In this heterogeneous case, we can find accurate values of *T_0_* numerically. As shown in Figure 1B, if *R_0_* is close to 1, host heterogeneity (*k*) does not really matter (*T_0_* tends to be high). However, if the pathogen has a high *R_0_* and thus spreads well, then host heterogeneity strongly affects *T_0_*. Additionally, we find that the heterogeneous threshold simply scales as a function of *k* and *R_0_^2^* (see Box 2). As an example, if *k* = 0.16, as estimated for SARS infections [11], the number of infected individuals needed to guarantee an outbreak increases 4-fold compared to a homogeneous population (Material S.3 in Text S1).

**Box 2. Heterogeneous Outbreak Threshold**

In a heterogeneous host population (see the main text for the bases of this heterogeneity), it has been shown that the number of secondary infections generated per infected individual can be well described by a negative binomial distribution with mean *R_0_* and dispersion parameter *k* [11]. The dispersion parameter determines the level of variation in the number of secondary infections: if *k* = 1, we have a homogeneous outbreak, but heterogeneity increases as *k* drops below 1; that is, it enlarges the proportion of infected individuals that are either “super-spreaders” or “dead-ends” (those that do not transmit the pathogen). Lloyd-Smith et al. [11] showed how to estimate *R_0_* and *k* from previous epidemics through applying a maximum-likelihood model to individual transmission data.

Although in this case it is not possible to find a strict analytical form for the outbreak threshold, progress can be made if we measure the ratio of the heterogeneous and homogeneous thresholds. This function yields values that are independent of a strict cutoff probability (Material S.3 in Text S1). By investigating this ratio, we first found that for a fixed *R_0_*, a function of order 1/*k* fitted the numerical solutions very well. By measuring these curves for different *R_0_* values, we further found that a function of order 1/*R_0_* ^2^ provided a good fit to the coefficients. By fitting a function of order 1/*kR_0_* ^2^ to the numerical data using least-squares regression in *Mathematica* 8.0 [19], we obtained the following adjusted form for the outbreak threshold *T_0_* in a heterogeneous population:
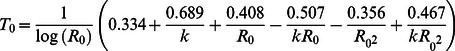
(1)As in the homogeneous case, *T_0_* only provides us with an order of magnitude and it can be multiplied by −Log(*c*) to find the number of infected hosts required for there to be a probability of outbreak equal to 1-*c*. A sensitivity analysis shows that Equation 1 tends to be more strongly affected by changes in *R_0_* than in *k* (Material S.3 in Text S1).

The outbreak threshold *T_0_* of an epidemic, which we define as the number of infected hosts above which there is very likely to be a major outbreak, can be estimated using simple formulae. Currently, to declare that an outbreak has occurred, studies choose an arbitrary low or high threshold depending, for instance, on whether they are monitoring disease outbreaks or modeling probabilities of emergence. We show that the outbreak threshold can be defined without resorting to an arbitrary cutoff. Of course, the generality of this definition has a cost, which is that the corresponding value of *T_0_* is only an order of magnitude. Modifications are needed to set a specific cutoff value or to capture host heterogeneity in transmission or incomplete sampling.

These results are valid if there are enough susceptible individuals present to maintain an epidemic in the initial stages, as assumed in most studies on emergence [6], [11]–[13], otherwise the pathogen may die out before the outbreak threshold is reached (Box 3 and Material S.2 in Text S1). Yet the key message generally holds that while the number of infections lies below the threshold, there is a strong chance that the pathogen will vanish without causing a major outbreak. From a biological viewpoint, unless *R_0_* is close to one, these thresholds tend to be small (on the order of 5 to 20 individuals). This contrasts with estimates of the Critical Community Size, which tend to lie in the hundreds of thousands of susceptible individuals [3], [7], [8]. Therefore, while only a small infected population is needed to trigger a full-scale epidemic, a much larger pool of individuals are required to maintain an epidemic, once it appears, and prevent it from fading out. This makes sense, since there tends to be more susceptible hosts early on in the outbreak than late on.

**Box 3. Effect of Limiting Host Population Size**

The basic result for the homogeneous population, *T_0_*∼1/Log(*R_0_*), assumes that during the time to pathogen outbreak, there are always enough susceptible individuals available to transmit to, so *R_0_* remains approximately constant during emergence. This assumption can be violated if *R_0_* is close to 1, or if the population size is small. More precisely, if the maximum outbreak size in a Susceptible-Infected-Recovered (SIR) epidemic, which is given by
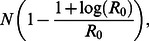
is less than 1/Log(*R_0_*), then the threshold cannot be reached. Since this maximum is dependent on the population size, outbreaks in smaller populations are less likely to reach the outbreak threshold. For example, if *N* = 10,000 then *R_0_* needs to exceed 1.06 for 1/Log(*R_0_*) to be reached; this increases to 1.34 if *N* decreases to 100. Further details are in Material S.2 in Text S1.

Estimates of *R_0_* and *k* from previous outbreaks can be used to infer the approximate size of this threshold, to determine whether a handful or hundreds of infected individuals are needed for an outbreak to establish itself. Box 4 outlines two case studies (smallpox in England and SARS in Singapore), estimates of *T_0_* for these, and how knowledge of the threshold could have aided their control. These examples highlight how the simplicity and rigorousness of the definition of *T_0_* opens a wide range of applications, as it can be readily applied to specific situations in order to determine the most adequate policies to prevent pathogen outbreaks.

**Box 4. Two Case Studies: Smallpox in England and SARS in Singapore**

A smallpox outbreak (Variola minor) was initiated in Birmingham, United Kingdom in 1966 due to laboratory release. We calculate a threshold such that the chance of extinction is less than 0.1%, which means that *T_0_* is equal to 7 times Equation 1. With an estimated *R_0_* of 1.6 and dispersion parameter *k* = 0.65 [11], *T_0_* is approximately equal to 9 infections. The transmission chain for this outbreak is now well-known [20]. Due to prior eradication of smallpox in the United Kingdom, the pathogen was not recognised until around the 45^th^ case was detected, by which point a full-scale epidemic was underway. A second laboratory outbreak arose in 1978, but the initial case (as well as a single secondary case) was quickly isolated, preventing a larger spread of the pathogen. Given the fairly low *T_0_* for the previous epidemic, early containment was probably essential in preventing a larger outbreak.

The SARS outbreak in Singapore in 2003 is an example of an outbreak with known super-spreaders [21], with an estimated initial *R_0_* of 1.63 and a low *k* of 0.16 [11]. *T_0_* is estimated to be around 27 infections. The first cases were observed in late February, with patients being admitted for pneumonia. Strict control measures were invoked from March 22^nd^ onwards, including home quarantining of those exposed to SARS patients and closing down of a market where a SARS outbreak was observed. By this date, 57 cases were detected, although it is unclear how many of those cases were still ongoing on the date. This point is important, as it is the infected population size that determines *T_0_*.

Overall, very early measures were necessary to successfully prevent a smallpox outbreak due to its rapid spread. In theory, it should have been “easier” to contain the SARS outbreak, as its threshold is three times greater than that for smallpox due to higher host heterogeneity (*k*). However, the first reported infected individual was a super-spreader, who infected at least 21 others. This reflects that in heterogeneous outbreaks, although the emergence probability is lower, the disease spread is faster (compared to homogeneous infections) once it does appear [11]. Quick containment of the outbreak was difficult to achieve since SARS was not immediately recognised, as well as the fact that the incubation period is around 5 days, by which point it had easily caused more secondary cases. However, in subsequent outbreaks super-spreaders might not be infected early on, allowing more time to contain the spread.

For newly-arising outbreaks, *T_0_* can be applied in several ways. If the epidemic initially spreads slowly, then *R_0_* and *T_0_* can be measured directly. Alternatively, estimates of *T_0_* can be calculated from previous outbreaks, as outlined above. In both cases, knowing what infected population size is needed to guarantee emergence can help to assess how critical a situation is. More generally, due to the difficulty in detecting real-world outbreaks that go extinct very quickly, experimental methods might be useful in determining to what extent different levels of *T_0_* capture the likelihood of full epidemic emergence.

## Supporting Information

Text S1 PDF file containing the following: **Material S.1:** Full derivations of outbreak threshold formulae for a homogeneous outbreak; **Material S.2:** Calculations of limitations due to small host population sizes; **Material S.3:** Finding solutions for outbreak threshold formulae for heterogeneous outbreaks.
(PDF)

Text S2 Same as Text S1, but in Mathematica format.
(ZIP)

## References

[1] Morens DM, Folkers GK, Fauci AS (2004) The challenge of emerging and re-emerging infectious diseases. Nature 430: 242–249. 15241422 10.1038/nature02759 PMC7094993

[2] Woolhouse M, Scott F, Hudson Z, Howey R, Chase-Topping M (2012) Human viruses: discovery and emergence. Philos Trans R Soc Lond B Biol Sci 367: 2864–2871. 22966141 10.1098/rstb.2011.0354 PMC3427559

[3] Fargette D, Konate G, Fauquet C, Muller E, Peterschmitt M, et al (2006) Molecular ecology and emergence of tropical plant viruses. Annu Rev Phytopathol 44: 235–260. 16784403 10.1146/annurev.phyto.44.120705.104644

[4] Anderson RM, May RM (1991) Infectious diseases of humans. Dynamics and control. Oxford: Oxford University Press. 757 p.

[5] Diekmann O, Heesterbeek JAP (2000) Mathematical epidemiology of infectious diseases: model building, analysis and interpretation. Chichester: John Wiley. 303 p.

[6] Allen L (2008) An introduction to stochastic epidemic models. In: Brauer F, van den Driessche P, Wu J, editors. Mathematical epidemiology. Berlin/Heidelberg: Springer. pp. 81–130.

[7] Bartlett MS (1960) The critical community size for measles in the United States. J R Stat Soc Ser A Stat Soc 123: 37–44.

[8] Keeling MJ, Rohani P (2007) Modelling infectious diseases in humans and animals. Princeton: Princeton University Press. 408 p.

[9] Antia R, Regoes RR, Koella JC, Bergstrom CT (2003) The role of evolution in the emergence of infectious diseases. Nature 426: 658–661. 14668863 10.1038/nature02104 PMC7095141

[10] Yates A, Antia R, Regoes RR (2006) How do pathogen evolution and host heterogeneity interact in disease emergence? Proc Biol Sci 273: 3075–3083. 17015347 10.1098/rspb.2006.3681 PMC1679899

[11] Lloyd-Smith JO, Schreiber SJ, Kopp PE, Getz WM (2005) Superspreading and the effect of individual variation on disease emergence. Nature 438: 355–359. 16292310 10.1038/nature04153 PMC7094981

[12] Kubiak RJ, Arinaminpathy N, McLean AR (2010) Insights into the evolution and emergence of a novel infectious disease. PLoS Comput Biol 6: e1000947 doi:10.1371/journal.pcbi.1000947 20941384 10.1371/journal.pcbi.1000947 PMC2947978

[13] Ferguson NM, Fraser C, Donnelly CA, Ghani AC, Anderson RM (2004) Public health risk from the avian H5N1 influenza epidemic. Science 304: 968–969. 15143265 10.1126/science.1096898

[14] Kaplan NL, Hudson RR, Langley CH (1989) The “hitchhiking effect” revisited. Genetics 123: 887–889. 2612899 10.1093/genetics/123.4.887 PMC1203897

[15] Barton NH (2000) Genetic hitchhiking. Philos Trans R Soc Lond B Biol Sci 355: 1553–1562. 11127900 10.1098/rstb.2000.0716 PMC1692896

[16] Desai MM, Fisher DS (2007) Beneficial mutation-selection balance and the effect of linkage on positive selection. Genetics 176: 1759–1798. 17483432 10.1534/genetics.106.067678 PMC1931526

[17] Galvani AP, May RM (2005) Epidemiology: dimensions of superspreading. Nature 438: 293–295. 16292292 10.1038/438293a PMC7095140

[18] Alexander HK, Day T (2010) Risk factors for the evolutionary emergence of pathogens. J R Soc Interface 7: 1455–1474. 20410190 10.1098/rsif.2010.0123 PMC2935601

[19] Wolfram Research, Inc. (2010) Mathematica Edition: Version 8.0. Champaign, Illinois: Wolfram Research, Inc.

[20] Shooter RA (1980) Report of the investigation into the cause of the 1978 Birmingham smallpox occurrence. London: HM Stationery Office. 231 p.

[21] Leo YS, Chen M, Heng BH, Lee CC, Paton N, et al (2003) Severe acute respiratory syndrome — Singapore, 2003. MMWR Morb Mortal Wkly Rep 52: 405–411. 12807088

[22] Gillespie DT (1977) Exact stochastic simulation of coupled chemical reactions. J Phys Chem 81: 2340–2361.

