# Correction: Introducing the Outbreak Threshold in Epidemiology

**DOI:** 10.1371/journal.ppat.1006415

**Published:** 2017-05-31

**Authors:** Matthew Hartfield, Samuel Alizon

In Box 4, the listed *T*_*0*_ values for the smallpox and SARS outbreaks are incorrect. In paragraph 1 of Box 4, “*T*_*0*_ is approximately equal to 9 infections” should read “*T*_*0*_ is approximately equal to 19 infections.” In addition, in paragraph 2 of Box 4, “*T*_*0*_ is estimated to be around 27 infections” should read “*T*_*0*_ is estimated to be around 54 infections.”
