# Supplementary material for: Introducing the Outbreak Threshold in Epidemiology
Source: PLoS Pathog. 2013 Jun 6;9(6):e1003277. doi: 10.1371/journal.ppat.1003277 (PMC3680036; doi:10.1371/journal.ppat.1003277)
Supplement: Text S1 — PDF file containing the following: Material S.1: Full derivations of outbreak threshold formulae for a homogeneous outbreak; Material S.2: Calculations of limitations due to small host population sizes; Material S.3: Finding solutions for outbreak threshold formulae for heterogeneous outbreaks. (PDF) [file ppat.1003277.s001.pdf]

Introducing the Outbreak Threshold

Matthew Hartfield & Samuel Alizon

Correspondance to **matthew.hartfield@ird.fr**.

Supplementary Material S.1: The outbreak threshold in a homogeneous population

The basic idea is that in a finite host population of size N, an outbreak has occured when enough infected individuals are present so that the probability of pathogen extinction due to stochastic loss is essentially zero. As a framework for investigating this, we will use a basic 'susceptible-infected-removed' (SIR) or 'susceptible-infected-susceptible' (SIS) type of infection [Anderson and May 1991]. The process of interest is the rate of change of infected individuals over time, which in both these models is equal to:

$$\frac{dI}{dt} = \left(\beta \frac{S}{N} - \delta\right) I$$

Where β is the contact rate, $1/\delta$ the mean duration of an infection, and S the total number of susceptible individuals at time t. If the whole population is susceptible, then $S = N$, and so the initial growth rate is equal to $\beta/\delta = R_0$. This is the reproductive ratio of the infection, the number of secondary infections caused by a single infected host in a population that is entirely susceptible [Anderson and May 1991].

In a stochastic model, the probability of extinction is equal to $\delta/\beta = 1/R_0$ [Grimmett and Stirzaker 2001, Hubbarde *et al.* 2007]. Furthermore, if we assume that the population size is large enough so that $S \simeq N$ as the outbreak is emerging (that is, there are enough susceptible hosts present to carry and spread a potential epidemic), then each infected individual has a reproductive ratio approximately equal to R_0. Therefore, if there are I_t infected individuals present at time t, the overall probability of extinction of the entire pathogen population approximately equals $(1/R_0)^{I_t}$ [May *et al.* 2001, Allen 2008].

We want to find the value of $I_t = T_0$ so that the probability of extinction drops below a certain threshold, c. To find this we need to solve $(1/R_0)^{T_0} = R_0^{-T_0} = c$, which is easily done:

```
Solve[R0^-T0 == c, T0]
```

Solve::ifun : Inverse functions are being used by Solve, so some
solutions may not be found; use Reduce for complete solution information. ≫

$$\left\{\left\{T_0 \to -\frac{\text{Log}[c]}{\text{Log}[R_0]}\right\}\right\}$$

This highlights the main result that **on the order of 1 / Log (R_0) infected individuals are needed to maintain an infection in a homogeneous population.**

We are also interested in finding a general result for T_0, which does not depend on a cutoff value c. We can show this asymptotically. Ideally we want to find a solution to $R_0^{-T_0} = 0$, but a solution does not exist. We can find an approximate solution by writing $T_0 = N\,\xi$, and solving to find the proportion of individuals needed ξ, if $\xi << 1$. Taking a Taylor series around $\xi = 0$:

```
Normal[Series[R0^-N ξ, {ξ, 0, 1}]]
```

$1 - N\,\xi\,\text{Log}[R_0]$

Solving this equal to zero gives $T_0 = N\xi = 1/\text{Log}(R_0)$, highlighting how **at least this many infected individuals are needed to guarantee an outbreak.** Note that this derivation implicitly assumes that N is finite; if N goes to infinity then it is clear that $R_0^{-N\xi}$ goes to zero, so no solution to ξ exists. This makes sense, since in such an

infinite population an outbreak will not go extinct if $I = 1$ and $R_0 > 1$, as in an infinite population the only sufficient condition for emergence is that R_0 exceeds 1. Therefore, it would not be possible to solve for ξ needed to push the extinction probability below a threshold, as the only solution would be zero [Anderson and May 1991].

This general result highlights that if the number of infected individuals reaches the order of $1/\mathrm{Log}(R_0)$, the extinction probability of the pathogenic population becomes low and approaches zero, especially if this limit is greatly exceeded. Threfore using $1/\mathrm{Log}(R_0)$ as the scaling for the outbreak threshold is appropriate.

This definition of the outbreak threshold has the great advantage of being general (it does not require a specific cutoff) but that this generality is traded-off against its specificity. The value of the outbreak threshold should always be seen as an order of magnitude. If one would want to apply the outbreak threshold as a strict threshold, one would be using an extinction probability equal to $c = e^{-1} \simeq 0.37$. This illustrates that once this asymptotic level has been reached, the pathogen population is likely to persist, but still has a sizeable chance of extinction. As such, this limit should be considered a ‘lower bound’ for the outbreak threshold in a homogeneous outbreak. If a more rigorous threshold is desired, then a multiple of $1/\mathrm{Log}(R_0)$ should be adopted, or $-\mathrm{Log}(c)/\mathrm{Log}(R_0)$ should be used to produce an exact threshold.

Note that if R_0 is approximately just greater than one, i.e. $R_0 \simeq 1 + \nu$ for $\nu << 1$, then $\mathrm{Log}(R_0)$ is approximately equal to:

```
Normal[Series[Log[1 + ν], {ν, 0, 1}]]
```

ν

Then the number of infecteds needed for an outbreak to occur is approximately equal to $1/\nu = 1/(R_0 - 1) = \delta/(\beta\text{-}\delta)$. Thus one can make parallels with classic population genetics results demonstrating that a new allele of fitness $1 + s$ needs to produce on the order of $1/s$ copies of itself before it is guaranteed to fix [Kaplan *et al.* 1989, Barton 2000, Desai and Fisher 2007].

■ Accounting for imperfect samping

If the sampling of an outbreak is imperfect, then only an average proportion p of all infected individuals are detected at a given time. This factor can be incorporated into the model.

If there are I_t actual infected individuals at time t, the probability that I_d infected individuals are detected is sampled from a Binomial distribution with parameters (I_t, p). Thus the mean number sampled at a certain time is equal to $p \simeq I_t$. So if the threshold T_0 has been reached, then the actual number of infected individuals detected equals $p \simeq T_0$, which is $p/\mathrm{Log}(R_0)$ at the lowest limit.

■ Time taken to reach threshold

If the number of infected hosts is negligible compared to that of susceptible hosts ($S \simeq N$) for the initial stages of an epidemic, then we can approximate the epidemic growth rate by:

$$\frac{dI}{dt} = (\beta - \delta)\, I$$

Therefore the number of infected individuals will grow approximately exponentially, with $I_t = I_0\, e^{(\beta-\delta)\,t}$, where I_0 is the initial number of infected individuals. By setting $I_t = T_0$, we find that the time that T_0 is reached by equals:

```
Solve[T0 == I0 Exp[(β - δ) t], t]
```

Solve::ifun : Inverse functions are being used by Solve, so some
solutions may not be found; use Reduce for complete solution information. ≫

$$\left\{\left\{t \to \frac{\mathrm{Log}\left[\frac{T_0}{i_0}\right]}{\beta - \delta}\right\}\right\}$$

Here, I_0 is usually close to 1, and unless R_0 is very close to one, $\mathrm{Log}(T_0)$ is of order 1 as well. Therefore it would take a duration of the order of $1/(\beta\text{-}\delta)$ to reach the threshold limit.

■ Delayed Sampling

In some cases there can exist a time lag τ between an individual becoming infected and that infection becoming apparent. This delay could arise due to several reasons, such as a delay in the infection being reported, or if there

is a latent period before the infection becomes apparent in a host. In this case, whilst still assuming that there is no limitation of susceptible hosts (S $\simeq$ N), the detected number of infected individuals at time t is equal to $I_0 e^{(\beta-\delta)(t-\tau)} = I_0\, e^{(\beta-\delta)\,t}\, e^{-(\beta-\delta)\,\tau} = I_t\, e^{-(\beta-\delta)\,\tau}$.

If the actual number of infected individuals I_t equals T_0, the observed number of individuals equals $T_0\, e^{-(\beta-\delta)\,\tau}$, and so the observed threshold has to be altered accordingly. However, this is still of order $1/\mathrm{Log}\,(R_0)$, unless the time lag τ is large.

Supplementary Material S.2: Effect of the limitation in host population size

The homogeneous results assume that during the emergence time of the pathogen, there are enough susceptibles present to transmit a pathogen to, so the overall reproductive rate is always approximately equal to R_0. However, if R_0 is close to one, or if the population size is small, then the assumption that the reprodutive rate each generation will remain constant will no longer be valid. This section explores when this assumption will break down.

In an SIR infection, the rate of change of the susceptible population is:

$$\frac{\mathrm{dS}}{\mathrm{dt}} = -\beta \frac{S}{N}$$

Combined with the term for dI/dt as above, we can create a term for the change in the number of infected individuals as a function of the susceptible population, by diving the two derivatives:

$$\frac{\mathrm{dI}}{\mathrm{dS}} = \frac{N\,\delta}{S\,\beta} - 1 = \frac{N}{S\,R_0} - 1$$

From this we can see that the rate of change equals zero at $S = N/R_0$, which is the point when the outbreak would reach its maximum peak and would start dying out. If this maximum value is less than the outbreak threshold, then it is clear that our previous assumptions are violated. By solving the above differential equation we find that:

```
DSolve[{Inf'[S] == (N δ)/(S β) - 1, Inf[N] == 0}, Inf[S], S]
```

```
{{Inf[S] → (N β - S β - N δ Log[N] + N δ Log[S])/β}}
```

Therefore the maximum number of infecteds that arises over an SIR epidemic is equal to this function, evaluated at S = N δ/β:

```
(N β - S β - N δ Log[N] + N δ Log[S])/β /. S → (N δ)/β
```

```
(N β - N δ - N δ Log[N] + N δ Log[(N δ)/β])/β
```

This simplifies to:

```
MaxI[ND_, R_] := ND (1 - (1 + Log[R])/R)
```

For $R = \beta/\delta$. By plotting the minimum threshold $1/\mathrm{Log}(R_0)$ against this maximum value of the infection, we can see when R_0 becomes large enough so that this minimum threshold can be reached in theory. The following plot is for N = 10,000:

```
EmergeHom[R_] := 1 / Log[R]
```

```
Plot[{EmergeHom[R], MaxI[10 000, R]}, {R, 1.02, 1.1},
 PlotStyle → {Thickness[0.01], Thickness[0.01]},
 AxesLabel → {R0, "Infected Individuals"}]
```

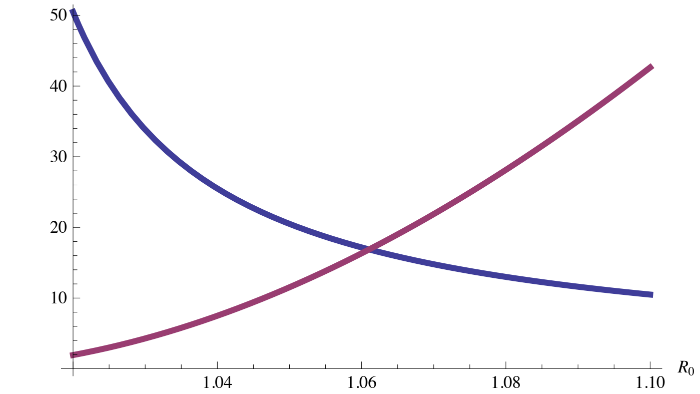

The red section denotes the values of R_0 for which the maximum number of infecteds is less than the outbreak threshold $1/\text{Log}[R_0]$. In theory, this means that the pathogen will go extinct before it reaches enough copies to guarantee not going extinct by stochastic drift alone, so the threshold cannot be reached due to a violation of the assumption that there are enough susceptibles at the start of an outbreak. The orange section denotes where the maximum number of infecteds exceeds the outbreak threshold, so the occurence of an outbreak can be detected using this threshold. For this particular case, R_0 needs to exceed 1.06 for the drift threshold to be reached (this of course depends on the value of N, the host population size).

For very small population sizes, R_0 needs to be rather large in order for an outbreak to be detected in a homogeneous population. If we repeat the plot for $N = 100$, for example:

```
Plot[{EmergeHom[R], MaxI[100, R]}, {R, 1.02, 1.5},
 PlotStyle → {Thickness[0.01], Thickness[0.01]},
 AxesLabel → {R0, "Infected Individuals"}]
```

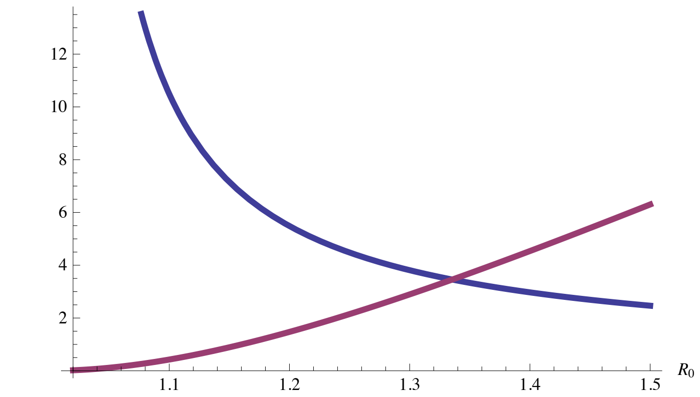

In this case, R_0 needs to exceed around 1.34 for the threshold to be reached.

Supplementary Material S.3: Finding a numerical approximation for the threshold in a heterogeneous population

■ Deriving the formulae

In a heterogeneous population, the probability of extinction is the solution s to the following:

$$\left(1 + \frac{R_0}{k}(1 - s)\right)^{-k} = s$$

Unfortunately, this is unsolveable for general k:

```
Solve[(1 + R/k (1 - s))^-k - s == 0, s]
```

Solve::nsmet : This system cannot be solved with the methods available to Solve. ≫

```
Solve[(1 + R (1 - s)/k)^-k - s == 0, s]
```

So instead we set up a function to numerically compute how many infected individuals are needed to equate the extinction probability to a threshold *c*.

```
FR3[R_, k_, c_] :=
 Inf /. FindRoot[(s /. FindRoot[0 == (1 + R/k (1 - s))^-k - s, {s, 0}])^Inf - c == 0, {Inf, Log[R]}]
```

By plotting this function for a range of *k*-values (corresponding to those found by maximum-likelihood analysis for several epidemics in Lloyd-Smith *et al*. [2005]), we see the general behaviour that the threshold decreases with increased R_0, and increases with lower *k* as the probability of extinction becomes higher, and thus more individuals are needed to guarantee an outbreak (see also Figure 1B in the main text).

```
LogPlot[{FR3[R, 0.16, Exp[-1.0]], FR3[R, 0.25, Exp[-1.0]], FR3[R, 0.35, Exp[-1.0]],
  FR3[R, 0.65, Exp[-1.0]], FR3[R, 1, Exp[-1.0]]}, {R, 1.01, 5},
 AxesOrigin → Automatic, PlotStyle → Thickness[0.005], AxesLabel → {R0, Threshold}]
```

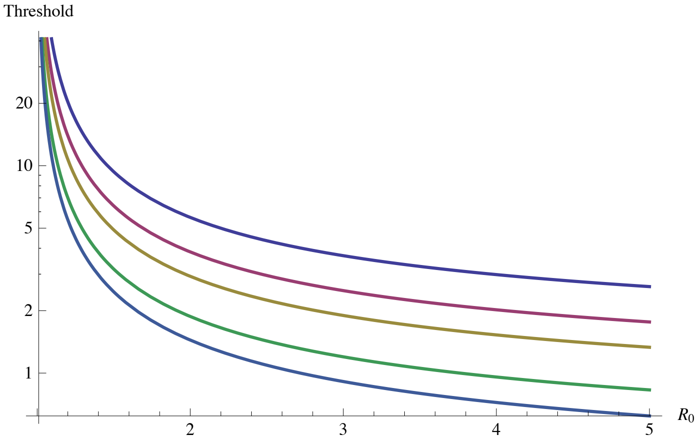

To proceed with finding an approximation, we first note that the ratio of the heterogeneous threshold with the homogeneous threshold is independent of the cutoff value *c*. To see this, let the extinction probability for an infected individual in a heterogeneous outbreak be equal to *p*. Now if we solve to find T_0 that reduces the overall extinction probability below a cutoff *c:*

```
Solve[p^T0 == c, T0]
```

Solve::ifun : Inverse functions are being used by Solve, so some
solutions may not be found; use Reduce for complete solution information. ≫

$$\left\{\left\{T_0 \to \frac{\mathrm{Log}[c]}{\mathrm{Log}[p]}\right\}\right\}$$

Therefore the ratio of the homogeneous threshold to the general threshold is independent of *c*:

$$\left(\frac{\mathrm{Log}[c]}{\mathrm{Log}[p]}\right) \Big/ \left(-\frac{\mathrm{Log}[c]}{\mathrm{Log}[R0]}\right)$$

$$-\frac{\mathrm{Log}[R0]}{\mathrm{Log}[p]}$$

Greater insight can be gained if we plot these thresholds, relative to those obtained for the same cutoff *c* in a homogeneous population. In this case, we see that the main scaling appears to be for *k* and only seems strongly dependent on R_0 when it gets closer to one.

```
HomThresh[R_, c_] := -Log[c]/Log[R]
```

```
Plot[{FR3[R, 0.16, 0.01] / HomThresh[R, 0.01], FR3[R, 0.25, 0.01] / HomThresh[R, 0.01],
  FR3[R, 0.35, 0.01] / HomThresh[R, 0.01], FR3[R, 0.65, 0.01] / HomThresh[R, 0.01],
  FR3[R, 1, 0.01] / HomThresh[R, 0.01]}, {R, 1.0001, 5},
 AxesOrigin → {1, 0}, PlotStyle → Thickness[0.01], AxesLabel → {R0, Threshold}]
```

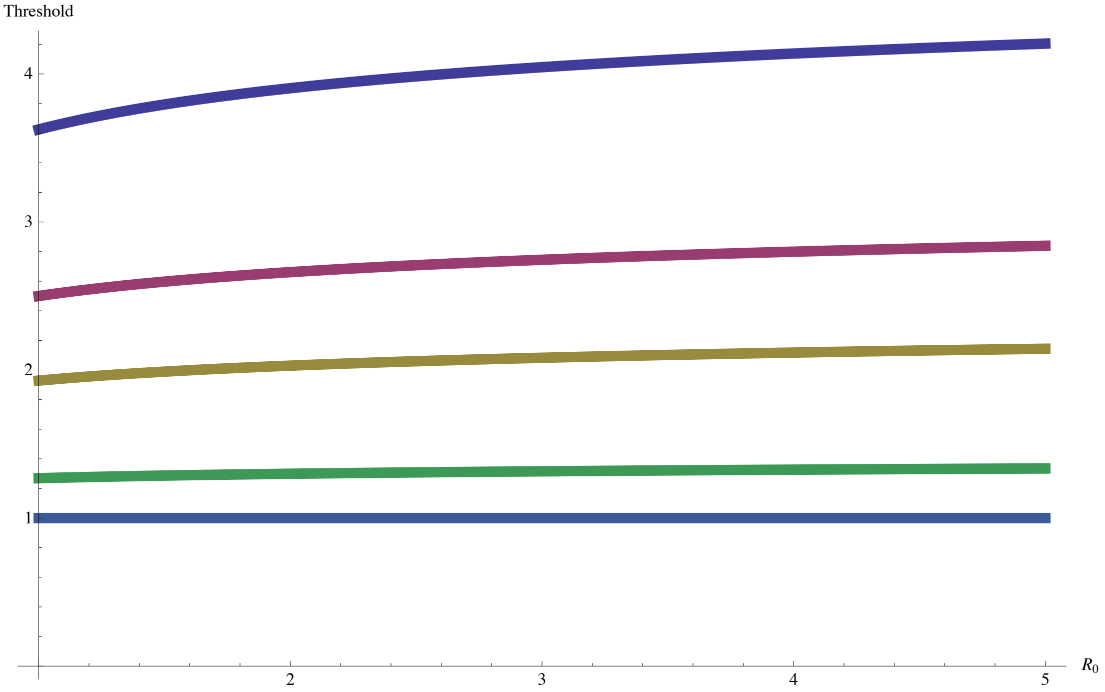

As a first approach, we observed that for a fixed R_0, it appears that the heterogeneous/homogeneous ratio scales according to a function of order $1/k$. As an example, here are the ratios if we set $R_0 = 5$.

```
{FR3[R, 0.1, 0.01] / HomThresh[R, 0.01], FR3[R, 0.16, 0.01] / HomThresh[R, 0.01],
  FR3[R, 0.25, 0.01] / HomThresh[R, 0.01], FR3[R, 0.35, 0.01] / HomThresh[R, 0.01],
  FR3[R, 0.65, 0.01] / HomThresh[R, 0.01], FR3[R, 1, 0.01] / HomThresh[R, 0.01]} /. R → 5
{6.47649, 4.20479, 2.83935, 2.14367, 1.33524, 1.}
```

The graph below shows a plot of these ratios, along with a fit of the curve $0.401 + \frac{0.608}{k}$, which provides a good match to the data. The coefficients were found using nonlinear regression analysis in R [R Development Core Team, 2008].

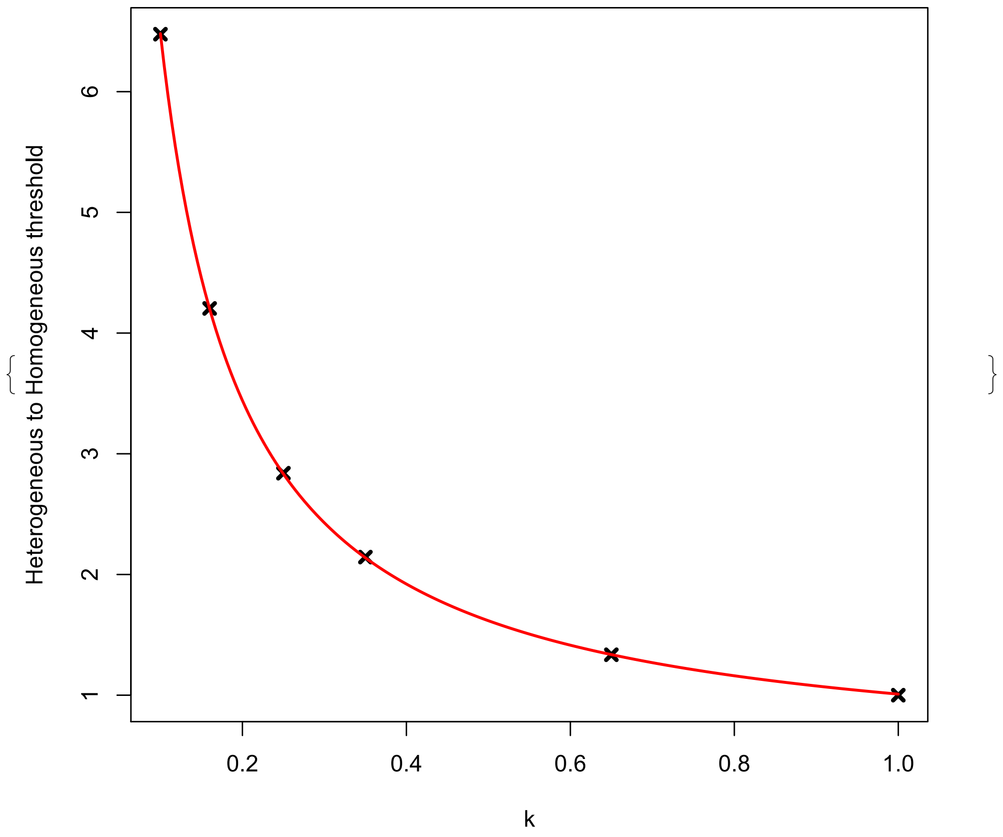

However, it is also clear that there is some dependence on R_0 on this threshold ratio as well. To try and find the form of this dependence, we repeated the above regression with $1/k$ but for different R_0 values. Then, we plotted the coefficients of the regression as a function of R_0, and determined what function appear to fit the data best. The below graphs show a plot of the coefficients, as well as a curve of the form $(a + b/R_0 + c/R_0)$, which appears to provide a good fit to data.

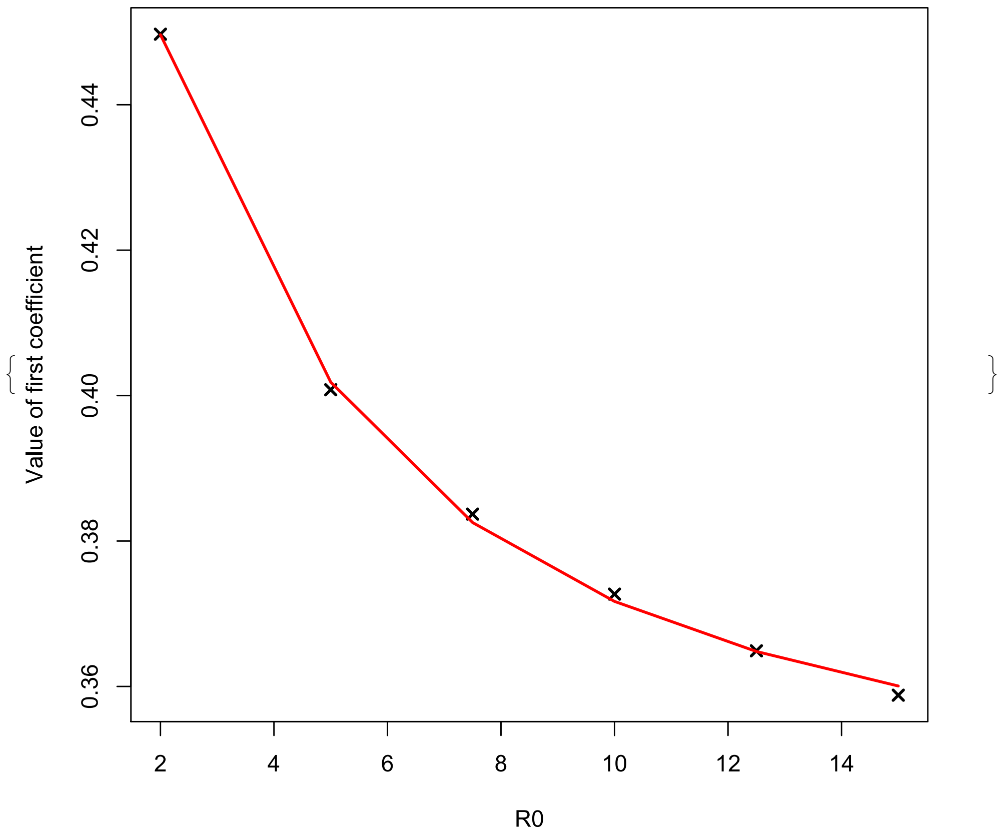
Value of first coefficient
0.36
0.38
0.40
0.42
0.44
2
4
6
8
10
12
14
R0

{ 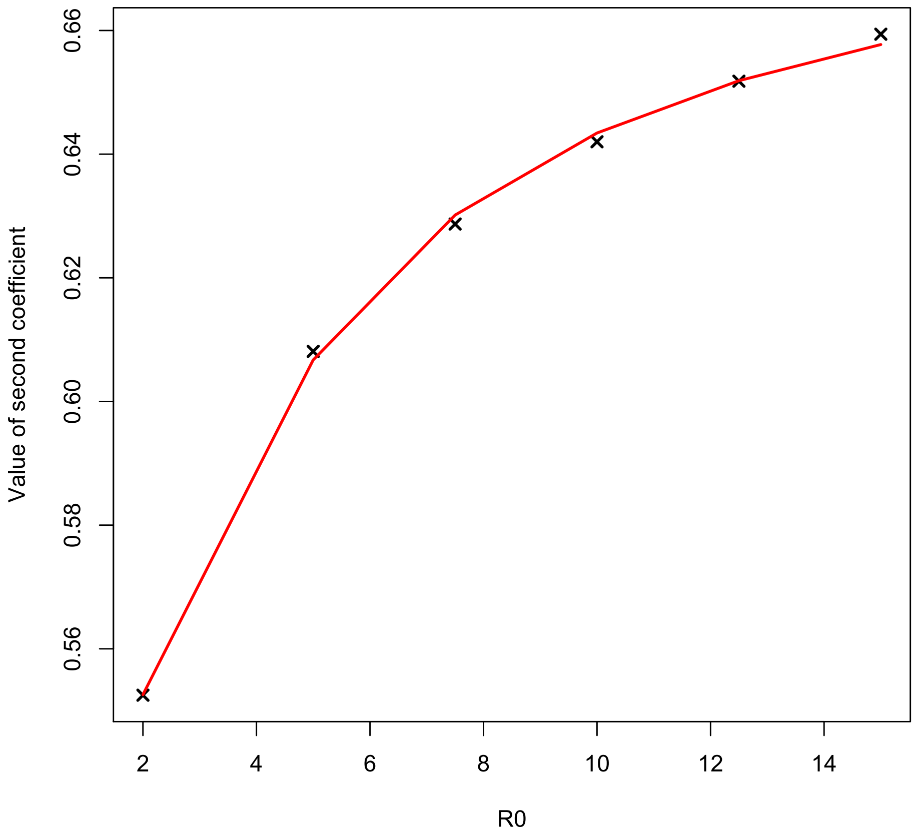 }

Combining both these regression curves, it appears that a function that provides a good fit to the heterogeneous/homogeneous ratio appear to takes the form:

$$\left(a + \frac{b}{k}\right)\left(c + \frac{d}{r} + \frac{e}{r^2}\right)$$

By generating numerical data for different R_0 and k values, we can use Mathematica's 'FindFit' function to find the coefficients of this function. Here's the data of the ratios for different R_0 values:

```
{FR3[R, 0.1, 0.01] / HomThresh[R, 0.01], FR3[R, 0.16, 0.01] / HomThresh[R, 0.01],
  FR3[R, 0.25, 0.01] / HomThresh[R, 0.01], FR3[R, 0.35, 0.01] / HomThresh[R, 0.01],
  FR3[R, 0.65, 0.01] / HomThresh[R, 0.01], FR3[R, 1, 0.01] / HomThresh[R, 0.01]} /. R → 2
```

```
{5.97335, 3.90369, 2.66125, 2.02961, 1.29937, 1.}
```

```
{FR3[R, 0.1, 0.01] / HomThresh[R, 0.01], FR3[R, 0.16, 0.01] / HomThresh[R, 0.01],
  FR3[R, 0.25, 0.01] / HomThresh[R, 0.01], FR3[R, 0.35, 0.01] / HomThresh[R, 0.01],
  FR3[R, 0.65, 0.01] / HomThresh[R, 0.01], FR3[R, 1, 0.01] / HomThresh[R, 0.01]} /. R → 5
```

```
{6.47649, 4.20479, 2.83935, 2.14367, 1.33524, 1.}
```

```
{FR3[R, 0.1, 0.01] / HomThresh[R, 0.01], FR3[R, 0.16, 0.01] / HomThresh[R, 0.01],
  FR3[R, 0.25, 0.01] / HomThresh[R, 0.01], FR3[R, 0.35, 0.01] / HomThresh[R, 0.01],
  FR3[R, 0.65, 0.01] / HomThresh[R, 0.01], FR3[R, 1, 0.01] / HomThresh[R, 0.01]} /. R → 7.5
```

```
{6.66327, 4.31758, 2.90689, 2.18744, 1.34941, 1.}
```

```
{FR3[R, 0.1, 0.01] / HomThresh[R, 0.01], FR3[R, 0.16, 0.01] / HomThresh[R, 0.01],
  FR3[R, 0.25, 0.01] / HomThresh[R, 0.01], FR3[R, 0.35, 0.01] / HomThresh[R, 0.01],
  FR3[R, 0.65, 0.01] / HomThresh[R, 0.01], FR3[R, 1, 0.01] / HomThresh[R, 0.01]} /. R → 10
```

```
{6.78469, 4.39114, 2.95112, 2.21622, 1.3588, 1.}
```

```
{FR3[R, 0.1, 0.01] / HomThresh[R, 0.01], FR3[R, 0.16, 0.01] / HomThresh[R, 0.01],
  FR3[R, 0.25, 0.01] / HomThresh[R, 0.01], FR3[R, 0.35, 0.01] / HomThresh[R, 0.01],
  FR3[R, 0.65, 0.01] / HomThresh[R, 0.01], FR3[R, 1, 0.01] / HomThresh[R, 0.01]} /. R → 12.5

{6.87313, 4.44483, 2.98348, 2.23733, 1.36571, 1.}

{FR3[R, 0.1, 0.01] / HomThresh[R, 0.01], FR3[R, 0.16, 0.01] / HomThresh[R, 0.01],
  FR3[R, 0.25, 0.01] / HomThresh[R, 0.01], FR3[R, 0.35, 0.01] / HomThresh[R, 0.01],
  FR3[R, 0.65, 0.01] / HomThresh[R, 0.01], FR3[R, 1, 0.01] / HomThresh[R, 0.01]} /. R → 15

{6.94195, 4.48666, 3.00874, 2.25381, 1.37113, 1.}
```

We can combine this into a single data and subsequently fit the model to it:

```
data = {{2, 0.1, 5.97335}, {2, 0.16, 3.90369},
  {2, 0.25, 2.66125}, {2, 0.35, 2.02961}, {2, 0.65, 1.29937} , {2, 1, 1},
      {5, 0.1, 6.47649}, {5, 0.16, 4.20479}, {5, 0.25, 2.83935},
  {5, 0.35, 2.14367}, {5, 0.65, 1.33524}, {5, 1, 1},
      {7.5, 0.1, 6.66327}, {7.5, 0.16, 4.31578}, {7.5, 0.25, 2.90689},
  {7.5, 0.35, 2.18744}, {7.5, 0.65, 1.34941}, {7.5, 1, 1},
      {10, 0.1, 6.78469}, {10, 0.16, 4.39114}, {10, 0.25, 2.95112},
  {10, 0.35, 2.21622}, {10, 0.65, 1.3588}, {10, 1, 1},
      {12.5, 0.1, 6.87313}, {12.5, 0.16, 4.44483}, {12.5, 0.25, 2.98348},
  {12.5, 0.35, 2.23733}, {12.5, 0.65, 1.36571}, {12.5, 1, 1},
      {15, 0.1, 6.94195}, {15, 0.16, 4.48666}, {15, 0.25, 3.00874},
  {15, 0.35, 2.25381}, {15, 0.65, 1.37113}, {15, 1, 1}}

{{2, 0.1, 5.97335}, {2, 0.16, 3.90369}, {2, 0.25, 2.66125}, {2, 0.35, 2.02961},
 {2, 0.65, 1.29937}, {2, 1, 1}, {5, 0.1, 6.47649}, {5, 0.16, 4.20479}, {5, 0.25, 2.83935},
 {5, 0.35, 2.14367}, {5, 0.65, 1.33524}, {5, 1, 1}, {7.5, 0.1, 6.66327},
 {7.5, 0.16, 4.31578}, {7.5, 0.25, 2.90689}, {7.5, 0.35, 2.18744}, {7.5, 0.65, 1.34941},
 {7.5, 1, 1}, {10, 0.1, 6.78469}, {10, 0.16, 4.39114}, {10, 0.25, 2.95112},
 {10, 0.35, 2.21622}, {10, 0.65, 1.3588}, {10, 1, 1}, {12.5, 0.1, 6.87313},
 {12.5, 0.16, 4.44483}, {12.5, 0.25, 2.98348}, {12.5, 0.35, 2.23733},
 {12.5, 0.65, 1.36571}, {12.5, 1, 1}, {15, 0.1, 6.94195}, {15, 0.16, 4.48666},
 {15, 0.25, 3.00874}, {15, 0.35, 2.25381}, {15, 0.65, 1.37113}, {15, 1, 1}}
```

$$\text{model} = a + \frac{b}{k} + \frac{c}{r} + \frac{d}{k\,r} + \frac{e}{r^2} + \frac{f}{k\,r^2}$$

$$a + \frac{b}{k} + \frac{e}{r^2} + \frac{f}{k\,r^2} + \frac{c}{r} + \frac{d}{k\,r}$$

```
fit = FindFit[data, model, {a, b, c, d, e, f}, {r, k}]

{a → 0.334401, b → 0.689435, c → 0.408427, d → -0.507054, e → -0.355933, f → 0.466701}
```

Therefore **the predicted function to fit the heterogeneous to homogeneous ratio is:**

$$\text{mfit}[r\_, k\_] := 0.334401 + \frac{0.689435}{k} + \frac{0.408427}{r} - \frac{0.507054}{k\,r} - \frac{0.355933}{r^2} + \frac{0.466701}{k\,r^2}$$

By comparing this function multiplied by the exact homogeneous threshold, we see that it provides a good fit to the numerical threshold calculated for heterogeneous outbreak. Here’s plot for k = 0.1, 0.5 and 0.9 for a c = 1% threshold:

```
plot1a = Plot[FR3[R, 0.1, 0.01], {R, 2, 15}, PlotStyle → {Red, Dashed}];
plot2a = Plot[FR3[R, 0.5, 0.01], {R, 2, 15}, PlotStyle → {Blue, Dashed}];
plot3a = Plot[FR3[R, 0.9, 0.01], {R, 2, 15}, PlotStyle → {Green, Dashed}];
```

```
Show[Plot[{mfit[x, 0.1] * Log[100.0]/Log[x]}, {x, 2, 15}, PlotStyle → {Red}], plot1a]
```

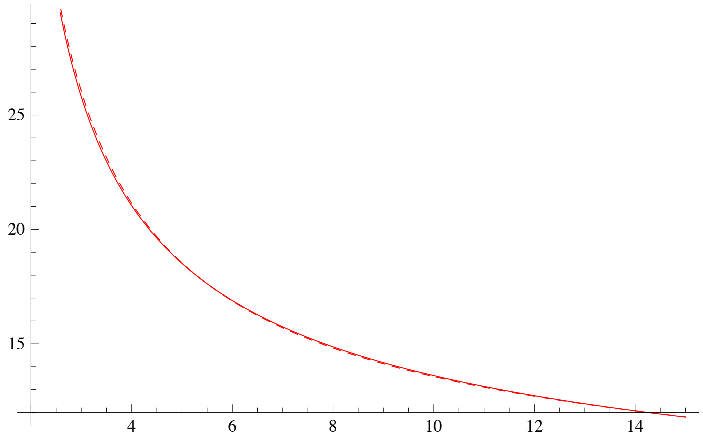


```
Show[Plot[{mfit[x, 0.5] * Log[100.0]/Log[x]}, {x, 2, 15}, PlotStyle → {Blue}], plot2a]
```

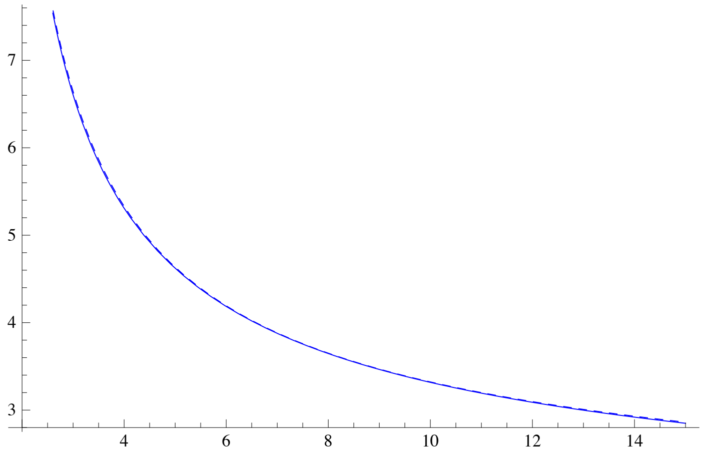


```
Show[Plot[{mfit[x, 0.9] * Log[100.0]/Log[x]}, {x, 2, 15}, PlotStyle → {Green}], plot3a]
```

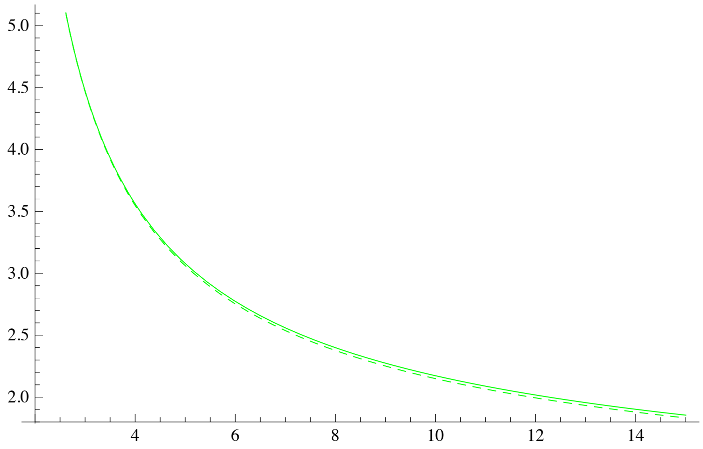

■ Sensitivity analysis

■ Sensitivity analysis

We next perform a sensitivity analysis of this function, in order to determine when the outbreak threshold is mostly dominated by changes in R_0 and k respectively. Specifically, we invoke an elasticity analysis (Caswell 2001, section 9.2), to determine the sensitivity of this approximation based on proportional changes in R_0 and k. For our approximation function $f(R_0, k)$, the elasticitiy of R_0 at a given point $(R_0{}^*, k^*)$ is equal to:

$$\frac{\partial f}{\partial R_0{}^*} \cdot \frac{R_0{}^*}{f(R_0{}^*, k^*)}$$

Sensitivity for R_0. The following figure shows the sensitivity of f to R_0:

```
FR4[R_, k_] := 1/Log[R] * mfit[R, k]

ContourPlot[(D[FR4[R2, k2], R2] /. {R2 → R, k2 → k}) * (R/FR4[R, k]),
 {R, 1.01, 5}, {k, 0.1, 1}, FrameLabel → {R0, k},
 ContourLabels → Function[{x, y, z}, Text[Framed[z], {x, y}, Background → White]],
 ColorFunction → "RedBlueTones", ContourStyle → Black]
```

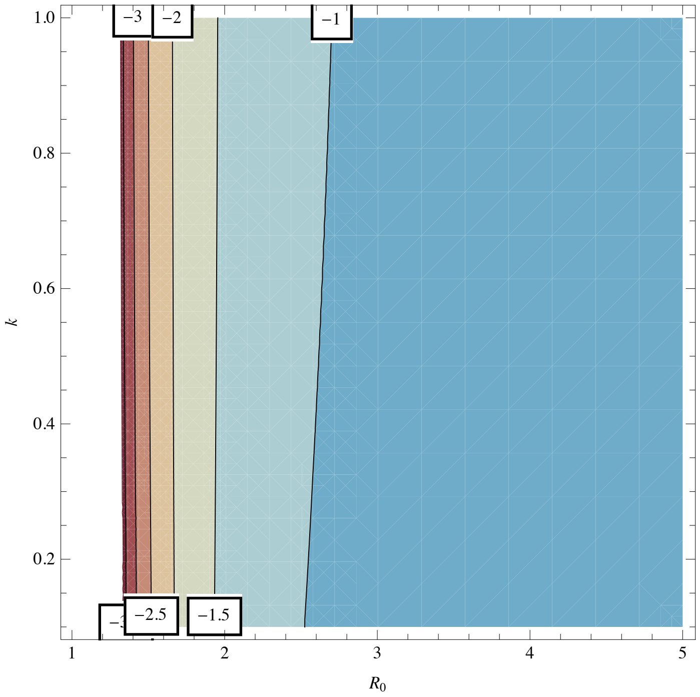

This plot shows that the sensitivity is negative and increases with higher R_0. This implies that as R_0 increases the change in threshold decreases, and does so at a slower rate for larger R_0. This is reflected in the $1/\text{Log}(R_0)$ function in the threshold approximation. Note however that for small R_0 close to one, the sensitivity becomes larger and negative, indicating a large increase in the number of infected indivduals needed for the outbreak to establish.

Sensitivity for k. Similarly, we can calculate the sensitivity of f to k:

```
ContourPlot[(D[FR4[R2, k2], k2] /. {R2 → R, k2 → k}) * (k / FR4[R, k]),
 {R, 1.01, 5}, {k, 0.01, 1}, FrameLabel → {R0, k},
 ContourLabels → Function[{x, y, z}, Text[Framed[z], {x, y}, Background → White]],
 ColorFunction → "RedBlueTones", ContourStyle → Black]
```

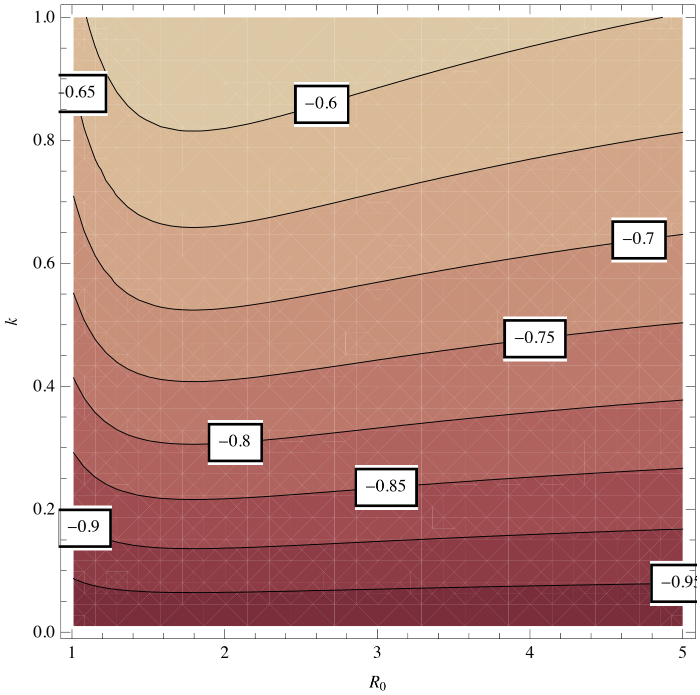

The sensitivity is negative and increases towards zero for larger k values, due to the $1/k$ behaviour of the outbreak threshold. Thus whilst sensitivity increases for smaller k, it does not cause as rapid an increase in the threshold level as changing R_0 by a similar proportion.

Bibliography for Supplementary Material

- Anderson RM, May RM (1991) Infectious Diseases of Humans. Dynamics and Control. Oxford: Oxford University Press.
- Grimmett GR, Stirzaker DR (2001) Probability and Random Processes. Oxford: Oxford University Press, third edition.
- Hubbarde JE, Wild G, Wahl LM (2007) Fixation probabilities when generation times are variable: The burst–death model. Genetics 176: 1703–1712.
- May RM, Gupta S, McLean AR (2001) Infectious disease dynamics: What characterizes a successful invader? Philos Trans R Soc Lond B Biol Sci 356: 899–910.
- Allen L (2008) An introduction to stochastic epidemic models. In: Brauer F, van den Driessche P, Wu J, editors, Mathematical Epidemiology, Berlin/Heidelberg: Springer, volume 1945 of Lecture Notes in Mathematics, chapter 3. pp. 81–130.
- Kaplan NL, Hudson RR, Langley CH (1989) The "hitchhiking effect" revisited. Genetics 123: 887–899.
- Barton NH (2000) Genetic hitchhiking. Philos Trans R Soc Lond B Biol Sci 355: 1553–1562.
- Desai MM, Fisher DS (2007) Beneficial mutation-selection balance and the effect of linkage on positive selection. Genetics 176: 1759–1798.
- Lloyd-Smith JO, Schreiber SJ, Kopp PE, Getz WM (2005) Superspreading and the effect of individual variation on disease emergence. Nature 438: 355– 359.
- R Development Core Team (2008). R: A Language and Environment for Statistical Computing. R Foundation for Statistical Computing, Vienna, Austria.

- Caswell H (2001) Matrix Population Methods. Sinauer Associates, 2nd edition.
